# Microbial community analysis of the hypersaline water of the Dead Sea using high‐throughput amplicon sequencing

**DOI:** 10.1002/mbo3.500

**Published:** 2017-07-05

**Authors:** Jacob H. Jacob, Emad I. Hussein, Muhamad Ali K. Shakhatreh, Christopher T. Cornelison

**Affiliations:** ^1^ Department of Biological Sciences Al al‐Bayt University Al‐Mafraq Jordan; ^2^ Department of Biological Sciences Yarmouk University Irbid Jordan; ^3^ Department of Medical Laboratory Sciences Jordan University of Science and Technology Irbid Jordan; ^4^ Division of Research and Advanced Study Kennesaw State University Kennesaw GA USA

**Keywords:** Dead Sea, metagenomics, microbial ecology

## Abstract

Amplicon sequencing using next‐generation technology (bTEFAP ^®^) has been utilized in describing the diversity of Dead Sea microbiota. The investigated area is a well‐known salt lake in the western part of Jordan found in the lowest geographical location in the world (more than 420 m below sea level) and characterized by extreme salinity (approximately, 34%) in addition to other extreme conditions (low pH, unique ionic composition different from sea water). DNA was extracted from Dead Sea water. A total of 314,310 small subunit RNA (SSU rRNA) sequences were parsed, and 288,452 sequences were then clustered. For alpha diversity analysis, sample was rarefied to 3,000 sequences. The Shannon–Wiener index curve plot reached a plateau at approximately 3,000 sequences indicating that sequencing depth was sufficient to capture the full scope of microbial diversity. Archaea was found to be dominating the sequences (52%), whereas Bacteria constitute 45% of the sequences. Altogether, prokaryotic sequences (which constitute 97% of all sequences) were found to predominate. The findings expand on previous studies by using high‐throughput amplicon sequencing to describe the microbial community in an environment which in recent years has been shown to hide some interesting diversity.

## 1 INTRODUCTION

The Dead Sea is a well‐known salt lake located in the western part of Jordan. This natural lake represents a unique habitat for extremophiles due to its extreme geophysicochemical characteristics. For instance, the Dead Sea surface is considered as the Earth's lowest elevation on land (more than 420 m below sea level) (Avriel et al., 2011). With respect to its salinity, the Dead Sea is characterized by extreme salinity, about 34%, which is approximately 10 times higher than the salinity of oceans (Bodaker et al., 2010). Moreover, the salinity in the Dead Sea is expected to increase in the upcoming years due to high evaporation and low regional precipitation. Former research articles have already reported that the negative water budget leads to a 1‐mol/L annual decrease in the Dead Sea level (Bodaker et al., 2010). In the Dead Sea water, magnesium is the dominant cation and the pH is low (around 6) (Oren, 2007). In addition, the Dead Sea area was found to have a unique UV radiation since UVA/UVB ratio is greater at the Dead Sea than anyplace else in the world (Avriel et al., 2011). Altogether, the geophysicochemical characteristics exemplify extreme conditions for living organisms and likely represent a unique microbial ecology.

In the last 5 years, several studies on samples taken from Dead Sea water, soil, and black mud were carried out to isolate and characterize the microorganisms flourishing in the Dead Sea area and to explore their biological activity (Jacob, 2012; Jacob & Irshaid, 2012, 2013; Jacob, Wink, & Wink, 2016). In these studies, the majority of isolates from the Dead Sea water were bacteria (Jacob, 2012). The isolated bacteria were members of both Gram‐positive bacteria (like *Arthrobacter* sp., *Kocuria erythromyxa*, and *Bacillus licheniformis*) and Gram‐negative bacteria (like *Salinivibrio costicola*,* Vibrio alginolyticus*, and *Chromohalobacter salexigens*). More recently, 13 morphologically different isolates of halophilic *Actinobacteria* from the soil and the black mud of the Dead Sea were isolated (Jacob et al., 2016). The isolates were found to be species of the genera *Brevibacterium*,* Rhodococcus*,* Leifsonia*,* Arthrobacter*,* Propionibacterium*,* Corynebacterium*, and *Trueperella*. Some isolates have a pronounced antimicrobial activity (Jacob et al., 2016).

These previous studies have utilized culture‐dependent methods to isolate and characterize the microorganisms in the Dead Sea. However, we believe that most of the microbial species are still unseen in this unusual area due to the limitations of the methods used, that is, the culture‐independent methods. Even though culture‐dependent methods are advantageous over culture‐independent, if a microbial material is required for further studies, the major disadvantage of culture‐dependent methods is that they are thought to undervalue the microbial number and composition of the studied samples (Al‐Awadhi et al., 2013).

In this study, we aim to explore the diversity of microbial species in the Dead Sea water using culture‐independent approach. Similar studies were conducted in the last years with certain types of samples from the Dead Sea. For instance, Rhodes, Oren, and House (2012) have investigated the 16S rRNA amplicon libraries from a Dead Sea haloarchaeal bloom in 1992 and compared it with the 2007 residual population and simulated blooms in experimental microcosms. Important population shifts were detected during the bloom, and unexpectedly a signature from the bloom was retained 15 years later. In another study, Bodaker et al. (2010) and Rhodes et al. (2012) analyzed the 16S rRNA genes from particulate matter obtained from a depth of 5 m at an offshore station via tangential filtration. Their results showed that members of the family Halobacteriaceae are existent in the water column. Analysis of 16S rRNA genes indicated that the Dead Sea, despite the harsh conditions to life, supports a unique and diverse community of halophilic Archaea (Bodaker et al., 2010; Rhodes et al., 2012).

In this current study, we explore the microbial communities in the Dead Sea water during June 2015 by metagenomic analysis. We aim in this study to determine which types of halophilic and/or halotolerant microbial taxa are present in the Dead Sea water by amplicon sequencing using the novel next‐generation technology (bTEFAP^®^). We also aim to determine the abundance of the detected taxa and the sequencing depth was evaluated to determine if it is sufficient to capture the full scope of microbial diversity. This study describes the new uncultivated species found in the Dead Sea water and represents a source data for future studies since this environment is changing continuously.

## 2 MATERIALS AND METHODS

### 2.1 Water sampling

Water samples (1 L) were collected in June 2015 from the surface water of the Dead Sea (31°36.545 N, 35°36.648 E). Geographic coordinates and elevation were determined using eTrex Legend C (GARMIN, Taiwan). Samples were collected in sterile clean glass bottles and transported directly to the laboratory for further processing and analysis.

### 2.2 Physicochemical analysis water

The in situ temperature and pH were determined by using the portable pH/temperature meter (Model TI 9000, WalkLAB, Trans Instruments, USA). Salinity was measured by a handheld salinity refractometer. The concentrations of Na, K, Ca, and Mg were determined using flame atomic absorption spectrometry (FAAS) (Varian Spectra AA, Australia). Air–acetylene flame was used. For each element, the measurements were made using its specific hollow cathode lamp. A stock solution (BDH chemicals Ltd., Poole, England) of each element was used to prepare the working standard solution for each element. The instrumental parameters were adjusted according to the manufacturer's recommendations (Obeidat, Al‐Momani, & Othman, 2016).

### 2.3 DNA extraction

Water samples were filtered through 0.2 μm membranes under vacuum. The membranes were then cut outv into small pieces. Pieces were then transferred to a 50 mL centrifuge tube. DNA extraction was then completed using E.Z.N.A.^®^ Water DNA kit (Omega Biotech Ltd., India) as per the manufacturer's instructions. The eluted DNA was stored at −20°C for further analysis.

### 2.4 Metagenome analysis

Amplicon sequencing was carried out via next‐generation technology (bTEFAP^®^) described earlier by Dowd, Sun, Wolcott, Domingo, and Carroll (2008) and it has been used in the analysis of wide‐ranging health and environmental samples (Dowd, Callaway, et al., 2008; Eren et al., 2011; Swanson et al., 2011). The 16S universal Eubacterial primers 515F GTGCCAGCMGCCGCGGTAA and 806R GGACTACHVGGGTWTCTAAT were used to evaluate the microbial ecology of the sample on the IlluminaMiSeq through methods via the bTEFAP^®^ DNA analysis service. PCR using HotStarTaq Plus Master Mix Kit (Qiagen, Valencia, CA) was carried out under the next conditions: 94°C for 3 min, afterward, 28 cycles of 94°C for 30 s; 53°C for 40 sec and 72°C for 1 min; then, the final elongation step was done at 72°C for 5 min. After PCR, amplicon products were mixed equally and purified using Agencourt Ampure beads (Agencourt Bioscience Corporation, MA, USA). Sequencing of samples was done via the IlluminaMiSeq chemistry according to the manufacturer's protocols. The Q25 sequence data generated from sequencing were processed by a proprietary analysis pipeline (www.mrdnalab.com, MR DNA, Shallowater, TX). Small sequences (less than 200 bp) were removed. Sequences were then denoised and chimeras were removed. After removal of singleton sequences, operational taxonomic units (OTUs) were defined, clustering at 97% similarity (3% divergence) (Dowd, Sun, et al. 2008; Dowd, Callaway, et al., 2008; Edgar, 2010; Eren et al., 2011; Swanson et al., 2011). Afterward, OTUs were classified by BLASTn against a curated GreenGenes/RDP/NCBI derived database (DeSantis et al., 2006) and assembled into each taxonomic level into “counts” as well as “percentage” files.

### 2.5 Statistical analysis

Statistical analysis was done utilizing various computer packages (NCSS, XLstat, 2007, “R,” and NCSS 2010). Alpha diversity examination was carried out as described previously (Dowd, Sun, et al. 2008; Dowd, Callaway, et al., 2008; Edgar, 2010; Eren et al., 2011; Swanson et al., 2011) using Qiime (www.qiime.org). Significance described for any analysis is defined as *p* < .05.

### 2.6 Alpha diversity

The number of OTUs at the species level was apprised to define alpha diversity among the different groups. Alpha diversity essentially evaluates how many unique bacterial species are within the sample.

## 3 RESULTS

### 3.1 Physicochemical analysis

The in situ temperature of the Dead Sea water sample was 22°C and the pH was 5.9. Salinity was found to be extremely high (34%). The concentration of Na, K, Ca, and Mg were also determined. Table 1 shows the concentration of the aforementioned elements in the Dead Sea water sample.

**Table 1:** The physicochemical properties of Dead Sea water and the concentration of some selected ions

Parameter/ion	Value/concentration
Salinity (in situ)	34%
pH (in situ)	5.9
Temperature (in situ)	22°C
Sodium	34.290 mg/L
Potassium	4.430 mg/L
Magnesium	46.100 mg/L
Calcium	23.770 mg/L

### 3.2 Metagenomic analysis

**Figure 1:**
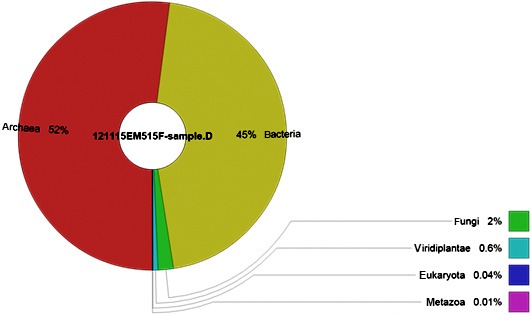
The microbial community composition in the Dead Sea water. Archaea and Bacteria are the major microbial groups with 97% abundance

After strict quality sequence curation, a total of 314,310 sequences were parsed and 288,452 were then clustered. Prokaryotic sequences (which constitute 97% of all sequences) were found to predominate and the sequences of Archaea were found to be more abundant than those of Bacteria (52% of the detected sequences belong to Archaea, whereas 45% belong to Bacteria; Figure 1). Minor abundance of eukaryotes was also detected (3%) (Figure 1).

**Figure 2:**
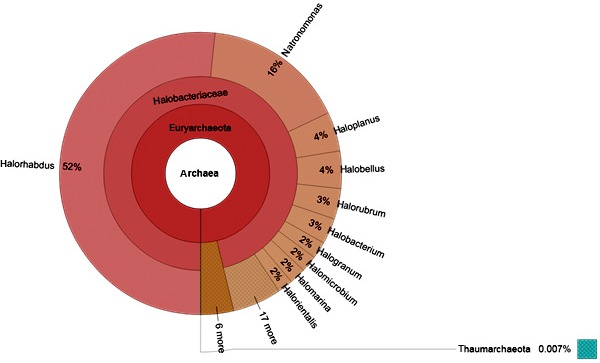
The composition of Archaeal population in the Dead Sea water. The dominant phylum is Euryarchaeota in which the family Halobacteriaceae is dominating. The most abundant genera in Halobacteriaceae are *Halorhabdus* (52% abundance) and *Natromonas* (16% abundance). Other genera with lower abundance are shown

After analyzing the Archaeal sequences, 100% of the sequences were found to belong to the phylum Euryarchaeota in which the family Halobacteriaceae is the dominant (Figure 2). The dominant genus in Halobacteriaceae was found to be *Halorhabdus* (52%) followed by the genus *Natronomonas* with 16% abundance. Other genera of Archaea were also detected, but with low abundance like *Haloplanus* (4%), *Halobellus* (4%), *Halorubrum* (3%), *Halobacterium* (3%), *Halogranum* (3%), *Halomicrobium* (3%), *Halomarina* (2%), and *Halorientalis* (2%) (Figure 2).

**Figure 3:**
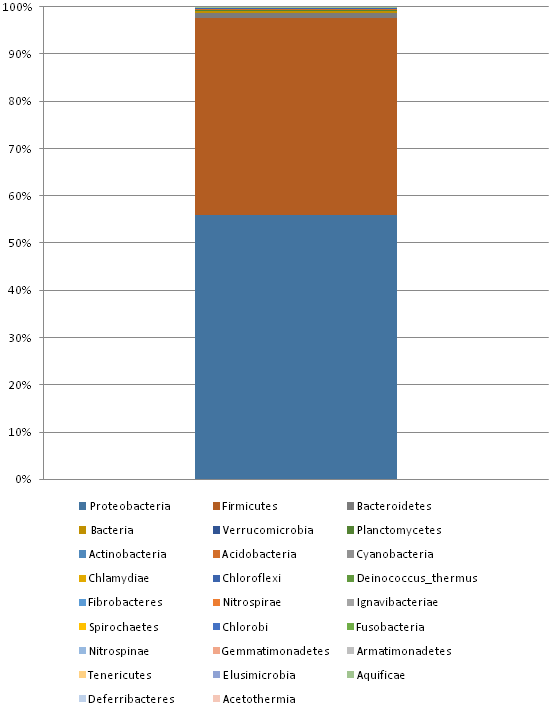
The composition of Bacterial population in the Dead Sea water. The most abundant bacterial phyla are Proteobacteria (more than 55% abundance) and Firmicutes (more than 41% abundance). Other phyla with lower abundance are also shown

**Figure 4:**
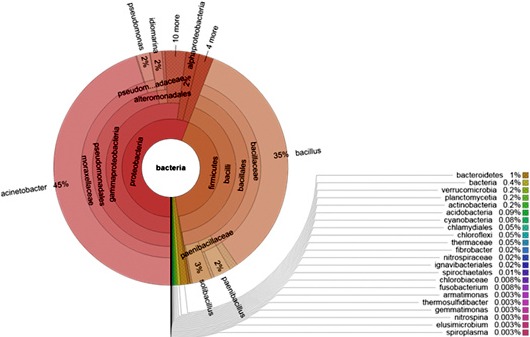
The main Bacterial genera in Dead Sea water and their relative abundance. Two main genera predominate: *Acinetobacter* (45% abundance) and *Bacillus* (35% abundance). Other genera were detected but with low relative abundance (3% or less)

On the other hand, sequences within the domain of Bacteria were found to constitute 45% of all sequences. Bacteria were dominated by two phyla: Proteobacteria (55.9%) and Firmicutes (41.7%) (Figure 3). Other phyla were also detected but with minor abundance like Bacteroidetes, Actinobacteria, and Cyanobacteria (Figure 3). With respect to dominant bacterial genera, two species were found to predominate: *Acinetobacter* (45%) and *Bacillus* (35%). The rest of genera constitute only 10% of the bacterial populations (Figure 4).

**Figure 5:**
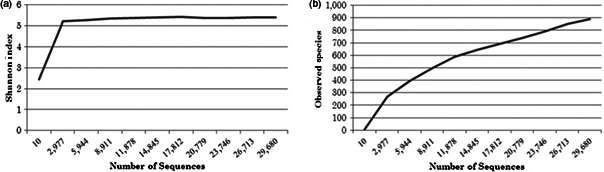
Shannon–Wiener index (a) and rarefaction curve (b). Shannon–Wiener index and rarefaction curve were calculated based on 97% sequence similarity

For alpha diversity analysis, sample was rarefied to 3,000 sequences. The Shannon–Wiener index curve plot (Figure 5) reaches a plateau at approximately 3,000 sequences indicating that sequencing depth was sufficient to capture the full scope of microbial diversity.

## 4 DISCUSSION

As revealed by the physicochemical analysis, the Dead Sea water is characterized by high salinity (34%), and therefore it was described as a typical example of a thalassohaline brine (Oren, 2007). Additionally, the pH was found to be relatively low (pH 5.9) indicating an acidic condition in water. The high salinity and acidic pH represent extreme conditions for living organisms. Moreover, some major cations were also determined in the Dead Sea water, namely Na, K, Mg, and Ca. The major cation that is present in highest concentration was found to be Mg, followed by Na, Ca, and K. This sequence of abundance agrees with the findings of other researchers who found that Mg, not Na, is the dominant cation in the Dead Sea water (Ma'or et al., 2006; Oren, 2007).

A metagenomic analysis of Dead Sea water was carried out by amplicon sequencing using next‐generation technology (bTEFAP^®^) to explore the diversity of microbial communities in the Dead Sea. Unsurprisingly, the dominant small subunit ribosomal RNA (SSU rRNA) gene sequences were found to belong to the domain of Archaea (52%) with less, but significant, percentage of bacterial amplicons. In a previous metagenomic analysis of samples collected from the Dead Sea after algal blooms in 1992 and 2007, Bodaker et al. (2010) found that most of the detected phylotypes were mostly related to Archaea (namely, the *Haloplanus nantans* [2007 bloom], *Halorhabdus utahensis* [2007 bloom], and *Halobacterium noricense* [1992 bloom]). However, in their metagenomic analysis, Bodaker et al. (2010) were unable to detect bacterial rRNA in their fosmid library which led them to conclude that this indicate the low quantity or even the absence of Bacteria in the Dead Sea. This conclusion does not agree with our findings because the number of bacterial species (i.e., species richness) in the Dead Sea was significant (SSU rRNA gene sequences identified within the domain of Bacteria constitute 45% of the detected species). This can be attributed to different techniques utilized to study the microbial community and the type of samples.

In our study, it was noted that the dominant Archaeal genus was *Halorhabdus* (52% of Archaea), followed by *Natronomonas* (12%), *Haloplanus* (4%), *Halobellus* (4%), *Halorubrum* (3%), *Halobacterium* (3%), *Halogranum* (3%), *Halomicrobium* (3%), *Halomarina* (2%), and *Halorientalis* (2%). In a metagenomic analysis carried out by Rhodes et al. (2012), it was concluded that Archaeal populations in the samples of Dead Sea were dominated by genera *Halosarcina* and *Natromonas* (Rhodes, 2012). Nevertheless, the microbial populations in the more recent sample (2007 sample) were dominated by *Halorhabdus* and *Natromonas*. The common dominant detected genus in the mentioned study and our study is the alkaliphilic genus *Natromonas*. Conversely, both *Halosarcina* and *Halorhabdus* were not detected in our study. It worth mentioning that these genera represent extreme halophiles that grow at high salinities (28%–34%NaCl), but most of them are unable to grow below 10%–15% NaCl (Häusler, 2014).

As mentioned earlier, Bacteria were also detected with a significant abundance (45%). The most abundant phyla were Proteobacteria (56%) and Firmicutes (42%). Minor abundances of the following phyla were also detected: Bacteroidetes (1%), Cyanobacteria (0.08%), Spirochaetes (0.01%). It is not surprising to detect diverse groups of bacteria in the Dead Sea since halophilic and halotolerant microorganisms are disseminated in the three domains of life. However, it is recorded that most of moderate and extreme halophiles are found in subgroups of Proteobacteria, Firmicutes, and in branches of Cyanobacteria, Bacteroidetes, and Spirochaetes (Häusler, 2014). This conclusion agrees with our results that showed that Proteobacteria and Firmicutes are the two major groups of Bacteria in the Dead Sea (both constitute 98% of bacterial sequences).

In this current study, we have also detected the minor abundance of eukaryotes in the Dead Sea water (only 3%). However, it should be noted that the primers used in this study (515F and 806R) are aiming at Bacteria and Archaea. Therefore, any eukaryotic sequence obtained is due to reaction with eukaryotic plastids, mitochondria, or chloroplast. Therefore, the eukaryotic sequence data were removed and were not considered.

This study highlights the ecological diversity that can be encountered in the Dead Sea as natural brine and serves to expand on previous studies that did not detect the significant bacterial community members of the Dead Sea. Because the Dead Sea is changing over time, additional studies to evaluate regional and seasonal changes in microbial populations of the Dead Sea are needed. Moreover, biomining of isolates are underway and should expand on the initial finding presented within.

## CONFLICT OF INTEREST

None declared.

## References

[mbo3500-bib-0001] Al‐Awadhi, H. , Dashti, N. , Khanafer, M. , Al‐Mailem, D. , Alii, N. , & Radwan, S. (2013). Bias problems in culture‐independent analysis of environmental bacterial communities: a representative study on hydrocarbonoclastic bacteria. Springer Plus, 2, 369 https://doi.org/10.1186/2193-1801-2-369 24040582 10.1186/2193-1801-2-369 PMC3769543

[mbo3500-bib-0002] Avriel, A. , Fuchs, L. , Plakht, Y. , Cicure, A. , Apfelbaum, A. , Satran, R. , … Sukenik, S. (2011). Quality of life at the Dead Sea region: the lower the better? An observational study. Health and Quality of Life Outcomes, 9, 38. 21615969 10.1186/1477-7525-9-38 PMC3123541

[mbo3500-bib-0004] Bodaker, I. , Sharon, I. , Suzuki, M. T. , Feingersch, R. , Shmoish, M. , Andreishcheva, E. , … Béjà, O. (2010). Comparative community genomics in the Dead Sea: an increasingly extreme environment. The ISME Journal, 4, 399–407. 20033072 10.1038/ismej.2009.141

[mbo3500-bib-0005] DeSantis, T. Z. , Hugenholtz, P. , Larsen, N. , Rojas, M. , Brodie, E. L. , Keller, K. , … Andersen, G. L. (2006). Greengenes, a chimera‐checked 16S rRNA gene database and workbench compatible with ARB. Applied and Environmental Microbiology, 72, 5069–5072. 16820507 10.1128/AEM.03006-05 PMC1489311

[mbo3500-bib-0006] Dowd, S. , Callaway, T. , Wolcott, R. , Sun, Y. , McKeehan, T. , Hagevoort, R. , & Edrington, T. (2008). Evaluation of the bacterial diversity in the feces of cattle using 16S rDNA bacterial tag‐encoded FLX amplicon pyrosequencing (bTEFAP). BMC Microbiology, 8, 125. 18652685 10.1186/1471-2180-8-125 PMC2515157

[mbo3500-bib-0007] Dowd, S. E. , Sun, Y. , Wolcott, R. D. , Domingo, A. , & Carroll, J. A. (2008). Bacterial tag‐encoded FLX amplicon pyrosequencing (bTEFAP) for microbiome studies: bacterial diversity in the ileum of newly weaned Salmonella‐infected pigs. Foodborne Pathogens and Disease, 5, 459–472. 18713063 10.1089/fpd.2008.0107

[mbo3500-bib-0008] Edgar, R. C. (2010). Search and clustering orders of magnitude faster than BLAST. Bioinformatics, 26, 2460–2461. 20709691 10.1093/bioinformatics/btq461

[mbo3500-bib-0009] Eren, A. , Zozaya, M. , Taylor, C. , Dowd, S. , Martin, D. , & Ferris, M. (2011). Exploring the diversity of *Gardnerella vaginalis* in the genitourinary tract microbiota of monogamous couples through subtle nucleotide variation. PLoS ONE, 6, e26732. 22046340 10.1371/journal.pone.0026732 PMC3201972

[mbo3500-bib-0010] Häusler, S. (2014). The environment, diversity and activity of microbial communities in submarine freshwater springs in the Dead Sea. Dissertation ZurErlangung des Grades einesDoktors der Naturwissenschaften – Dr. rer. nat. – dem Fachbereich Geowissenschaften der Universität Bremen.

[mbo3500-bib-0011] Jacob, J. (2012). Classification of halophilic heterotrophic bacteria thriving in the Jordanian Dead Sea littoral zone. Journal of Biological Sciences, 12, 246–252.

[mbo3500-bib-0012] Jacob, J. , & Irshaid, F. (2012). Biochemical and molecular taxonomy of a mild halophilic strain of Citrobacter isolated from hypersaline environment. Research Journal of Microbiology, 7, 219–226.

[mbo3500-bib-0013] Jacob, J. , & Irshaid, F. (2013). A New *Shewanella putrefaciens* strain isolated from the Dead Sea of Jordan. Malaysian Applied Biology Journal, 42, 1–7.

[mbo3500-bib-0014] Jacob, J. , Wink, J. , & Wink, M. (2016). Antimicrobial activity of actinobacteria from a hypersaline area of the Dead Sea. Proceedings of the Fourth Kuwait Conference of Chemistry Kuwait, March 20‐22, 2016 “Chemistry and Life Sciences”.

[mbo3500-bib-0015] Ma'or, Z. , Henis, Y. , Alon, Y. , Orlov, E. , Sørensen, K. , & Oren, A. (2006). Antimicrobial properties of Dead Sea black mineral mud. International Journal of Dermatology, 45, 504–511. 16700781 10.1111/j.1365-4632.2005.02621.x

[mbo3500-bib-0016] Obeidat, S. , Al‐Momani, I. , & Othman, R. (2016). Partitioning of trace metals in sediments from the Mediterranean coastal zone of Ajdabia to Benghazi, Libya: case study. Jordan Journal of Chemistry, 11, 247–258.

[mbo3500-bib-0017] Oren, A. (2007). Biodiversity in highly saline environments In Gerday C., & Glansdorf N. (Eds.), Physiology and biochemistry of extremophiles (pp. 223–231). Washington: ASM press.

[mbo3500-bib-0018] Rhodes, M. , Oren, A. , & House, C. (2012). Dynamics and persistence of Dead Sea microbial populations as shown by high‐throughput sequencing of rRNA. Applied and Environmental Microbiology, 78, 2489–2492. 22267671 10.1128/AEM.06393-11 PMC3302590

[mbo3500-bib-0019] Swanson, K. S. , Dowd, S. E. , Suchodolski, J. S. , Middelbos, I. S. , Vester, B. M. , Barry, K. A. , … Fahey, G. C. Jr (2011). Phylogenetic and gene‐centric metagenomics of the canine intestinal microbiome reveals similarities with humans and mice. ISME Journal, 5, 639–649. 20962874 10.1038/ismej.2010.162 PMC3105739

